# Antisense reduction in NADP‐ME in the C_4_ species *Flaveria bidentis* alters stomatal sensitivity to intercellular [CO_2_ ]

**DOI:** 10.1111/nph.71012

**Published:** 2026-02-18

**Authors:** Emmanuel L. Bernardo, Cristina Rodrigues Gabriel Sales, Tianshu Sun, Johannes Kromdijk

**Affiliations:** ^1^ Department of Plant Sciences University of Cambridge Downing Street CB23EA Cambridge UK; ^2^ Institute of Crop Science, University of the Philippines Los Baños College of Agriculture and Food Science Los Baños 4031 Laguna Philippines

**Keywords:** C_4_ photosynthesis, CO_2_ assimilation, *Flaveria bidentis*, NADP‐ME, photosynthesis, stomatal conductance

## Abstract

- Stomata have the crucial role of balancing the uptake of CO_2_ for photosynthesis and water loss via transpiration by adjusting their aperture. In C_3_ plants, coordination between photosynthesis and stomatal conductance can be disrupted by mutations in Calvin–Benson–Bassham (CBB) cycle expression, but it is not clear whether the disruption of the C_4_ carbon concentrating mechanism would have similar effects.
- In this study, it was hypothesized that perturbing the C_4_ cycle, resulting in reduced carbon flux into the bundle sheath cells and overall net CO_2_ assimilation rates, may alter stomatal regulation. To test this hypothesis, we performed gas exchange measurements on transgenic *Flaveria bidentis* plants carrying an antisense construct leading to reduced nicotinamide adenine dinucleotide phosphate (NADP)‐malic enzyme (NADP‐ME) activity.
- Stomatal conductance was unaltered in the antisense plants at ambient CO_2_ levels. However, C_i_ was significantly increased. When measurements were instead performed at common C_i_, stomatal conductance was significantly higher in the antisense lines.
- These results demonstrate that disruption of the C_4_ cycle via reduced NADP‐ME activity in *F. bidentis* leads to altered stomatal sensitivity to C_i_.

## Introduction

Stomatal pores on the leaf epidermis regulate gas exchange between plants and the atmosphere. By adjusting their aperture through guard cell turgor changes, stomata balance CO_2_ uptake for photosynthesis with water loss via transpiration. Plants must therefore continuously optimize stomatal aperture to maximize CO_2_ uptake while minimizing dehydration, which is achieved through dynamic responses to environmental and internal signals. In general, stomata open when conditions favor carbon assimilation and close under stress or excess CO_2_. For instance, stomatal apertures widen when exposed to low CO_2_ concentrations, high light, and high humidity but close under high CO_2_, darkness, low humidity, high temperature, and drought (Farquhar & Sharkey, 1982; Marshall *et al*., 1996; Fricker & Willmer, 2012). By making these coordinated adjustments, plants maintain efficient gas exchange and water balance essential for survival in changing environments.

One key regulator of stomatal opening and closing is the concentration of CO_2_ inside the leaf (intercellular CO_2_, C_i_). Guard cells are thought to respond to changes in C_i_ rather than CO_2_ (C_a_), forming a feedback loop between photosynthesis and stomatal conductance (Mott, 1988, 2009). An increase in C_i_ (as during the night or in CO_2_‐rich air) triggers guard cells to lose turgor and close, whereas a decrease in C_i_ (as during photosynthesis) induces stomatal opening. Although this mechanism is often described as guard cell autonomous, as shown by the fact that responses are maintained in epidermal peel experiments that lack mesophyll tissue (Fitzsimons & Weyers, 1986), subsequent studies indicate that mesophyll‐derived signals are required for complete stomatal responses to CO_2_ (Engineer *et al*., 2016).

Mesophyll cells can modulate stomatal behavior by producing diffusible metabolites, which can accumulate in the apoplast surrounding guard cells and relay information about the leaf's photosynthetic activity (Engineer *et al*., 2016; Taylor *et al*., 2025). For example, red light often induces stomatal opening even when C_i_ is held constant, demonstrating a C_i_‐independent response that likely involves photoreceptor or mesophyll‐derived signals (Messinger *et al*., 2006; Taylor *et al*., 2024). Thus, additional links between mesophyll photosynthesis and guard cell autonomous sensing pathways are apparent (Lawson *et al*., 2014), and guard cells do not only adjust to C_i_ but also integrate additional mesophyll‐derived signals or light‐dependent cues, collectively termed C_i_‐independent responses, to fine‐tune stomatal aperture. Meanwhile, C_i_‐dependent responses are those directly controlled by changes in intercellular CO_2_ concentration sensed by guard cells (Messinger *et al*., 2006; Lawson *et al*., 2008).

While coordination between stomatal conductance and photosynthetic rate is typically tight, they can be decoupled through transgenic manipulations. Previous studies in transgenic plants demonstrated that antisense reduction in Calvin–Benson–Bassham (CBB) cycle enzyme activities such as Rubisco (Furbank *et al*., 1996), phosphoribulokinase (Paul *et al*., 1995), glyceraldehyde‐3‐phosphate dehydrogenase (Price *et al*., 1995), or chloroplastic fructose‐1,6‐bisphosphatase (Muschak *et al*., 1999) can severely decrease photosynthetic capacity but with little or no change in stomatal conductance. For instance, tobacco plants with < 25% of normal Rubisco content maintained near‐normal *g*_s_ under high light (von Caemmerer *et al*., 2004). Across many of these studies, antisense plants exhibited elevated C_i_ levels without stomatal closure. However, while many of these studies reported observations of *g*_s_, the experiments were generally not designed to wait long enough for the stomatal responses to fully unfold nor account for indirect physiological effects on *g*_s_ such as increases in C_i_ when A decreases, which complicate the interpretation of stomatal responses in these studies (Lawson, 2009). Indeed, when SBPase antisense plants were compared under controlled C_a_, as is most common, *g*_s_ was found to be different, but so was C_i_ (Lawson *et al*., 2008). However, when *g*_s_ was measured under common C_i_, differences were no longer apparent. This demonstrates the importance of assessing the impact of such confounding effects. Furthermore, the degree to which the stomata open or close (and therefore *g*_s_ changes) depends on the stomatal anatomical and morphological characteristics, including stomatal density (SD, the number of stomata per leaf area), which can be further affected by the prevailing growth conditions (Casson & Gray, 2008; Torii, 2015) and may also be perturbed by antisense manipulations of CO_2_ fixation. Nevertheless, only a few studies investigating the impact of antisense reduction of *A*_n_ on stomatal conductance account for potential confounding effects of both C_i_ and stomatal patterning.

C_4_ plants provide an attractive experimental setup to evaluate the impact of antisense reduction in *A*_n_ on stomatal conductance for several reasons. First, because of the presence of the C_4_ carbon concentrating mechanism, *A*_n_ is typically (close to) CO_2_‐saturated in C_4_ plants (Pignon & Long, 2020). As a result, changes in *g*_s_ in response to experimentally varying C_a_ or C_i_ can be interpreted without the confounding effect of simultaneous impacts on *A*_n_. Second, adjusting the carbon uptake flux by perturbing C_4_ cycle activity should not directly affect guard cell photosynthesis, in contrast to many Calvin cycle mutants. Because of these reasons, mutants in C_4_ cycle enzymes avoid some of the common caveats to verify whether changes in mesophyll carbon flux modify stomatal behavior through C_i_‐dependent or C_i_‐independent mechanisms.

Here, we investigated stomatal regulation in transgenic C_4_ *Flaveria bidentis* plants with reduced nicotinamide adenine dinucleotide phosphate (NADP)‐malic enzyme (NADP‐ME) activity. In C_4_ plants, NADP‐ME is the key decarboxylase in the C_4_ cycle that converts malate to pyruvate leading to the release of CO_2_ in the chloroplasts of bundle sheath cells. Reducing NADP‐ME activity limits malate decarboxylation and delivery of nicotinamide adenine dinucleotide phosphate (NADPH) and CO_2_. We hypothesized that this perturbation of the C_4_ cycle would alter the coordination between CO_2_ assimilation and stomatal conductance by affecting either the C_i_ signal perceived by the guard cells or via C_i_‐independent signals linked to mesophyll carbon metabolism. While responses to red light and stomatal sensitivity to blue light were unaffected by the decrease in NADP‐ME activity, NADP‐ME antisense lines of *Flaveria bidentis* had higher operational C_i_ at ambient CO_2_ levels than wild‐type (WT). When additional measurements were performed under common C_i_, *g*_s_ was significantly increased in the antisense lines. These findings imply that stomatal movements in these plants are adjusted to maintain transpiration via altered stomatal sensitivity to C_i_. In addition, the lack of differences at ambient CO_2_ levels may suggest that the CO_2_ levels sensed by guard cells may include a contribution of C_a_.

## Materials and Methods

### Plant materials

WT and T_1_ seeds of NADP‐ME antisense *Flaveria bidentis* (L.) were provided by Prof. Susanne von Caemmerer (Australian National University, Canberra, Australia). The antisense construct used to generate the transgenic lines was originally described by Pengelly *et al*. (2010) and consisted of an 845‐bp fragment in antisense orientation from the central coding region of ChlME1, the primary chloroplastic C_4_ isoform of NADP‐ME identified in *F. trinervia* and *F. bidentis* (GenBank accession nos. X57142 and AY863144). The T‐DNA cassette contained the CaMV 35S promoter, the NADP‐ME antisense fragment, a Nos terminator, and a kanamycin resistance cassette for selection. Seven independent transformation events were generated, coded as 1A‐4, 1A‐5, 1A‐6, 1A‐7, 1A‐8, 2A‐1, and 2A‐2. Two out of three specific antisense events used in this study (1A‐5, 1A‐8, and 2A‐1) were independent from those previously characterized in Pengelly *et al*. (2012) and represent additional transgenic events derived from the same construct.

### PCR confirmation and copy number estimation

The presence of the NeoR/KanR selectable marker gene was confirmed via PCR using primers AATGAACTGCAGGACGAGGC and AATGAACTGCAGGACGAGGC. Copy number analysis was performed following Głowacka *et al*. (2016) using reverse transcription polymerase chain reaction (RT‐PCR) on genomic DNA with FbACTIN gene as a reference amplicon (AATGGAAGCTGCTGGTATTCA) and (CAACCACCTTGATCTTCATGC primers).

### Growth conditions

WT and T_1_ seeds were sown in plastic pots (10 cm H × 9 cm L × 9 cm W) filled with M3 compost (Levington®; Scotts, Ipswich, UK). The substrate was amended with controlled‐release fertilizer (Osmocote®; Scotts Miracle‐Gro Company, Marysville, OH, USA) at a rate of 1.0–1.5 g l^−1^ substrate. Plants were grown in controlled‐environment chambers (Conviron®; Controlled Environments Ltd, Winnipeg, MB, Canada) in a 12 h : 12 h, day : night cycle at 20°C, 65% RH, and a photon flux density (PFD) of 350 μmol m^−2^ s^−1^. The plants were hand‐watered to field capacity twice a week. PCR‐confirmed T_1_ plants carrying a single copy integration of the NADP‐ME antisense construct were self‐pollinated to obtain T_2_ seeds. T_2_ homozygous plants were grown under similar conditions as the T_1_ generation, but with a lower PFD of 150 μmol m^−2^ s^−1^, since plants exposed to 350 μmol m^−2^ s^−1^ showed leaf bleaching (Supporting Information Fig. S1) in the antisense plants. These growth conditions resulted in mutant plants, which looked identical to WT plants and grew at similar rates (Fig. S2). T_2_ plants were self‐pollinated, and the seeds were collected to generate T_3_ homozygous lines. All phenotyping was performed on 8‐wk‐old plants from T_3_ homozygous lines using the second or third recently expanded leaf.

### Protein and Chl content

Three replicate disks with a total area of 1.5 cm^2^ were collected using metal cork borers from recently expanded leaves from the same plants measured for gas exchange. Samples were flash‐frozen in liquid N_2_ and then stored at −80°C for further use. Frozen leaf samples (1.5 cm^2^) were homogenized in ice‐cold mortar and pestle into basic extraction buffer. Chls were extracted from 40 μl crude leaf extract in 960 μl 80% ethanol. The solution was incubated at 22°C (room temperature) in the dark for 2 h, and the absorbance was determined spectrophotometrically at 649 and 665 nm, allowing calculation of Chl content following Wintermans & De Mots (1965). Protein content was estimated against Bovine Serum Albumin (BSA) standards using Bradford assay (Sigma, Product No. B6916).

### SDS‐PAGE and immunoblot analysis

SDS‐PAGE was performed using 2 μg of total protein from each sample. Blotting of proteins to polyvinylidene difluoride (PVDF) membranes was performed using *Trans*‐Blot Turbo RTA Transfer Kit, PVDF (BIO‐RAD Laboratories Inc.) according to the manufacturer's instructions. Before assembly, the PVDF membrane was briefly activated with absolute methanol. Both the PVDF membrane and the filter paper were then soaked and equilibrated in 1× transfer buffer (diluted from the stock solution of Trans‐Blot Turbo 5× transfer buffer, BIO‐RAD Laboratories Inc. (Cat. No. 10026938)). Transblotting of western blot sandwich was performed using the Trans‐Blot Turbo Transfer System (BIO‐RAD Laboratories Inc., Hercules, CA, USA). All subsequent steps were performed on the blotted membranes under gentle agitation (60–100 rpm) using a horizontal shaker (Ultimax 1010; Heidolph Instruments, Schwabach, Germany). Membranes were first immersed in 1× Tris buffered saline (TBS) buffer (88 mM Tris base, 2.5 M NaCl, and adjusted to pH 7.5 with HCl) with sufficient volume covering the membrane. After washing, the membrane was blocked with 5% (w/v) nonfat milk in Tween‐TBS (TTBS) buffer (1× TBS, 0.05% (v/v) Tween‐20) for 60 min at room temperature. The membranes were then washed twice with TTBS for 5 min/wash, followed by primary antibody incubation overnight at 4°C in 1% (w/v) BSA/milk and TTBS. A primary antibody against NADP‐ME from *Zea mays* was used (courtesy of Robert Sharwood, Western Sydney University, Australia) at 1 : 2000 dilution.

Upon completion of the primary antibody incubation, the membrane was washed four times with TTBS for 5 min per wash. The membrane was then incubated with secondary antibody (HRP conjugated antibody (goat antirabbit IgG (H&L), HRP‐conjugated; product no. AS09602, 1 : 10 000 dilution, polyclonal antibody, Agrisera), 1% (w/v) BSA/milk and TTBS) for 1–2 h, followed by washing with TTBS three times and TBS two times at 5 min/wash. Lastly, the membrane was incubated with 7 ml of Clarity Western ECL Substrate (1 : 1 luminol/enhancer solution: peroxide chemiluminescent detection reagent; BIO‐RAD Laborotories Inc.) for 5 min before visualization on the transilluminator (G:BOX; Syngene) using the GeneSys software (v.1.7.2; Syngene). Subsequently, Coomassie blue staining (0.05% (w/v) Coomassie brilliant blue G‐250, 50% (v/v) methanol, 10% (v/v) glacial acetic acid and adjusted with ultrapure water) was carried out on the same membrane to confirm equal loading. The membrane was incubated with Coomassie blue staining solution for 5 min and stained with the staining solution (40% (v/v) methanol, 10% (v/v) glacial acetic acid), and destaining solution (40% (v/v) methanol, 10% (v/v) with ultrapure water) for 5–10 min. For visualization, the membrane was imaged with a transilluminator (G:BOX; Syngene) using the GeneSys software (v.1.7.2; Syngene, Cambridge, UK).

### Enzyme activity assays

Maximum NADP‐ME activity was determined following Pengelly *et al*. (2012) and Sharwood *et al*. (2016). Briefly, frozen leaf samples (1.5 cm^2^) were homogenized using ice cold mortar and pestle in 900 μl of basic ice‐cold extraction buffer containing 50 mM Bicine–NaOH, pH 8.2, 1 mM EDTA, 50 mM 2‐mercaptoethanol, 1 mM dithiothreitol, 1% (v/v) protease inhibitor cocktail, and 1% (w/v) polyvinylpyrrolidone in CO_2_‐sparged deionized H_2_O.

The homogenate was centrifuged at 13 000 ***g*** for 1 min, and the supernatant was used in the assay. For each assay, 100 μl of the supernatant was added to 900 μl of the NADP‐ME assay buffer (50 mM Bicine–NaOH, pH 8.2, 1 mM EDTA, 5 mM Malate) in a 96‐well microtiter plate. The reaction was initiated by adding 10 μl of 200 mM MgCl_2_. The activity of NADP‐ME was calculated by monitoring the decrease in absorbance of NADPH at 340 nm using a diode array spectrophotometer plate reader (Synergy HTX; Agilent Biotek).

Maximal phosphoenol pyruvate carboxylase (PEPC) activity was measured using an NADH‐linked assay as described in Sharwood *et al*. (2016). Frozen leaf samples (1.5 cm^2^) were homogenized in ice‐cold mortar and pestle with 600 μl of extraction buffer (50 mM Bicine–NaOH, pH 8.2, 1 mM EDTA, 50 mM 2‐mercaptoethanol, 1 mM dithiothreitol, 1% (v/v) protease inhibitor cocktail, and 1% (w/v) polyvinylpyrrolidone, CO_2_‐sparged deionized H_2_O). The homogenate was centrifuged for 1 min at 13 000 ***g***, and the supernatant was desalted on a Zeba™ spin desalting column (Thermo Scientific). To perform the assay, 5 μl of supernatant was added to 195 μl assay buffer (1 M HEPES‐NaOH, pH 8.0, 1 M MgCl_2_•6H_2_O, 0.1 M EDTA, 0.5 M NaHCO_3_, 0.1 M D‐glucose 6‐phosphate sodium salt, 8.5 KU mL^−1^ malic dehydrogenase (MDH), 14 mM β‐nicotinamide adenine dinucleotide (NADH, reduced disodium salt), and 0.2 M phospho(enol)pyruvic acid trisodium salt hydrate) and the reaction was initiated by the addition of 5 μl of 5 mM PEP. The activity was calculated by monitoring the decrease in NADH absorbance at 340 nm with a diode array spectrophotometer plate reader (Synergy HTX; Agilent Biotek, Winooski, VT, USA) after initiating the reaction.

Rubisco initial and total activity were measured from leaf extracts after Sales *et al*. (2020) with slight modifications. Leaf samples (1.5 cm^2^) were snap‐frozen in liquid nitrogen and stored at −80°C until use. They were then ground in an ice‐cold mortar and pestle with 1500 μl of extraction buffer containing 50 mM Bicine–NaOH, pH 8.2, 20 mM MgCl_2_, 1 mM EDTA, 2 mM benzamidine, 5 mM ε‐aminocaproic acid, 50 mM 2‐mercaptoethanol, 10 mM dithiothreitol, 1% (v/v) protease inhibitor cocktail (Sigma‐Aldrich), and 1 mM phenylmethylsulfonyl fluoride. The homogenate was clarified by centrifugation at 14 000 ***g*** and 4°C for 1 min. The supernatant was immediately used for measuring Rubisco activity at 25°C. For initial activity, the reaction was started by adding 5 μl homogenate to 195 μl complete assay buffer. For total activity, Rubisco in leaf extracts was first activated by incubation in the assay buffer containing CO_2_ and Mg^2+^ but in the absence of RuBP for 10 min in the dark, and then the reaction was initiated by adding 6 μl of 20 mM RuBP. The change in absorbance at 340 nm was monitored with a diode array spectrophotometer plate reader (Synergy HTX; Agilent Biotek) after initiating the reactions.

For all enzyme activity assays, measured absorbance values on the microplate plate reader (Synergy HTX; Agilent Biotek) were normalized to a path length of 1 cm (the typical cuvette width used in spectrophotometers (Burnett, 1972)), using a factor of 5.47 mm, considering both the volume and dimensions of the well (Sales *et al*., 2018). The reaction proceeded for at least 2 min to obtain a clear slope of decreasing absorbance. The first minute of the linear range of the activity was used to calculate the activities.

### Gas exchange measurements

All gas exchange measurements were performed using LI‐6400XT gas exchange system (LICOR Biosciences, Lincoln, NE, USA) on the youngest fully expanded leaf of 8‐wk‐old plants. Because blue light and C_i_ signals are known to converge (Hiyama *et al*., 2017), we decided to do a complete assessment of stomatal sensitivity to red/blue composition, as well as increasing red light intensity under constant C_i_. To characterize stomatal responses to differences in red and blue composition of incident light, leaves were first acclimated under 100% red light for at least 40 min. They were then switched to 75% red (630 nm) and 25% blue light (470 nm) for 60 min, followed by re‐exposure to the original light environment of 100% red light for another 60 min. A PPFD of 500 μmol m^−2^ s^−1^ was maintained throughout the experiment. The blue light treatment was initiated when steady state was reached (typically after 2 h). Gas exchange measurements were logged every 60 s throughout the protocol. Reference CO_2_ during the experiment was controlled at 400 μmol mol^−1^, block temperature was kept at 25°C, and average VPD was *c*. 1.2 kPa. All measurements were taken between 10:00 h and 16:00 h.

To further characterize the responses of stomatal conductance to red light intensity while keeping C_i_ constant, the protocol by Messinger *et al*. (2006) was adopted. This protocol measures the stomatal response to red light while controlling for the potentially confounding effect of the stomatal CO_2_ response by maintaining a constant intercellular CO_2_ concentration (C_i_) at each light intensity. Briefly, the Messinger protocol (Messinger *et al*., 2006) involved illuminating the leaf segment clamped inside the cuvette with 800 μmol m^−2^ s^−1^ red light to steady state. Subsequently, PFD was lowered stepwise from 800 to 600, 400, 200, 0 μmol m^−2^ s^−1^. Each change was initiated once steady‐state stomatal conductance had been reached, with at least a 40‐min waiting period. CO_2_ was manually adjusted using the mixer millivolt signal to keep C_i_ at the value observed at 800 μmol m^−2^ s^−1^.

### Stomatal morphology and density

To estimate stomatal morphology and density, epidermal imprints were taken from adaxial and abaxial layers using clear nail varnish (Rimmel, London, UK) using the same leaves used for gas exchange measurements. Dried nail varnish was lifted from the leaf using sticky tape (Sellotape®; Henkel Ltd, Hemel Hempstead, UK) and affixed onto glass slides. Images were captured using a Nikon microscope (Olympus BX41, Japan) fitted with a CCD camera (U‐TV0.5zc‐3/Micropublisher 3.3 RTV; Olympus, Tokyo) and installed with Q‐Capture Pro 7 imaging software. Total magnifications of ×200 and ×400 (Olympus WHB 10×/20 eyepiece × Olympus PN 20×/0.40 or PCN 60×/0.80 objectives) were used for SD counting and investigation of stomatal anatomical parameters, respectively.

To assess stomatal size (SS), guard cell width, and guard cell length were measured manually using the Fiji software (v.2.14.0; National Institutes of Health, Bethesda, MD, USA). SS was calculated using the formula for the area of an ellipse. SD was calculated as the number of stomata per unit area (mm^2^). A stomate was counted if > 50% was located within the field of view. Four random microscopic fields of view each from the adaxial and abaxial surfaces were collected from 3 to 5 biological replicates, giving > 300 stomata per species.

### Statistical analyses

To estimate the impact of red and blue light spectral composition on stomatal conductance, a linear mixed‐effects model was used. To perform the analysis, *g*_s_ and A at specific time points before each switch, and at the end of the R→RB→R experiment (*t* = 5 min, *t* = 65 min, and *t* = 125 min) were extracted from the gas exchange time series. The models included *Genotype* and *Light treatment* (R or RB) as fixed effects, with biological replicate (*Replicate*) specified as a random effect to account for repeated measurements.

For the red‐light response experiment, a separate linear mixed‐effects model was used with *Genotype* and red light photosynthetic PFD (*PPFD*) as fixed effects and *Replicate* as a random effect: *Response variable* ~ *Genotype* x *PFD* + (1|*Replicate*). Model assumptions were verified by inspecting residual distributions and using the Shapiro–Wilk test for normality. Models were fitted using restricted maximum likelihood via the lmer() function from the lme4 (v.1.1‐35.5; Bates *et al*., 2015) package in R (v.4.4.1; R Core Team, 2024). *Post hoc* pairwise comparisons among genotypes were performed on model‐estimated means using Tukey's multiplicity adjustment (Tukey–Kramer method) for all pairwise contrasts, implemented though emmeans (v.1.10,1; Lenth, 2024) and multcomp (v.1.4‐25; Hothorn *et al*., 2008), with an α level of 0.05.

We used a similar linear mixed‐effects model framework to evaluate the effect of genotype on enzyme activities, total soluble protein (TSP), and Chl content. The model included *Genotype* as a fixed effect and biological replication (*Replicate*) as a random effect: *Response variable* ~ *Genotype* + (1|*Replicate*). *Post hoc* pairwise comparisons were performed using Tukey's Honestly Significant Difference (HSD) test at α = 0.05.

The SD on the abaxial and adaxial surfaces was analyzed using a two‐way ANOVA, and Tukey's HSD (α = 0.05) was used to separate the means if a significant effect of genotype, leaf side, or their interaction was detected. Data on stomatal size were processed similarly.

## Results

### NADP‐ME activity is decreased by 66–73% in homozygous antisense *Flaveria bidentis* lines

**Fig. 1:**
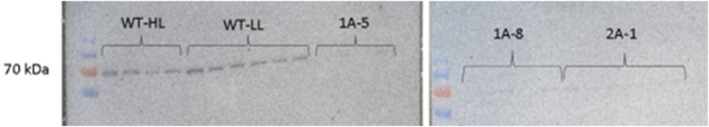
NADP‐malic enzyme (NADP‐ME) protein expression in wild‐type (WT) plants of *Flaveria bidentis* and plants expressing an antisense construct against NADP‐ME. The relative expression level of NADP‐ME in WT plants was evaluated when grown under regular light intensity (HL, 350 μmol m^−2^ s^−1^) and low light intensity (LL, 150 μmol m^−2^ s^−1^) to match the conditions of the antisense lines. The plants for three independent antisense lines (1A‐5, 1A‐8, and 2A‐1) were grown under the lower intensity to avoid light stress.

**Fig. 2:**
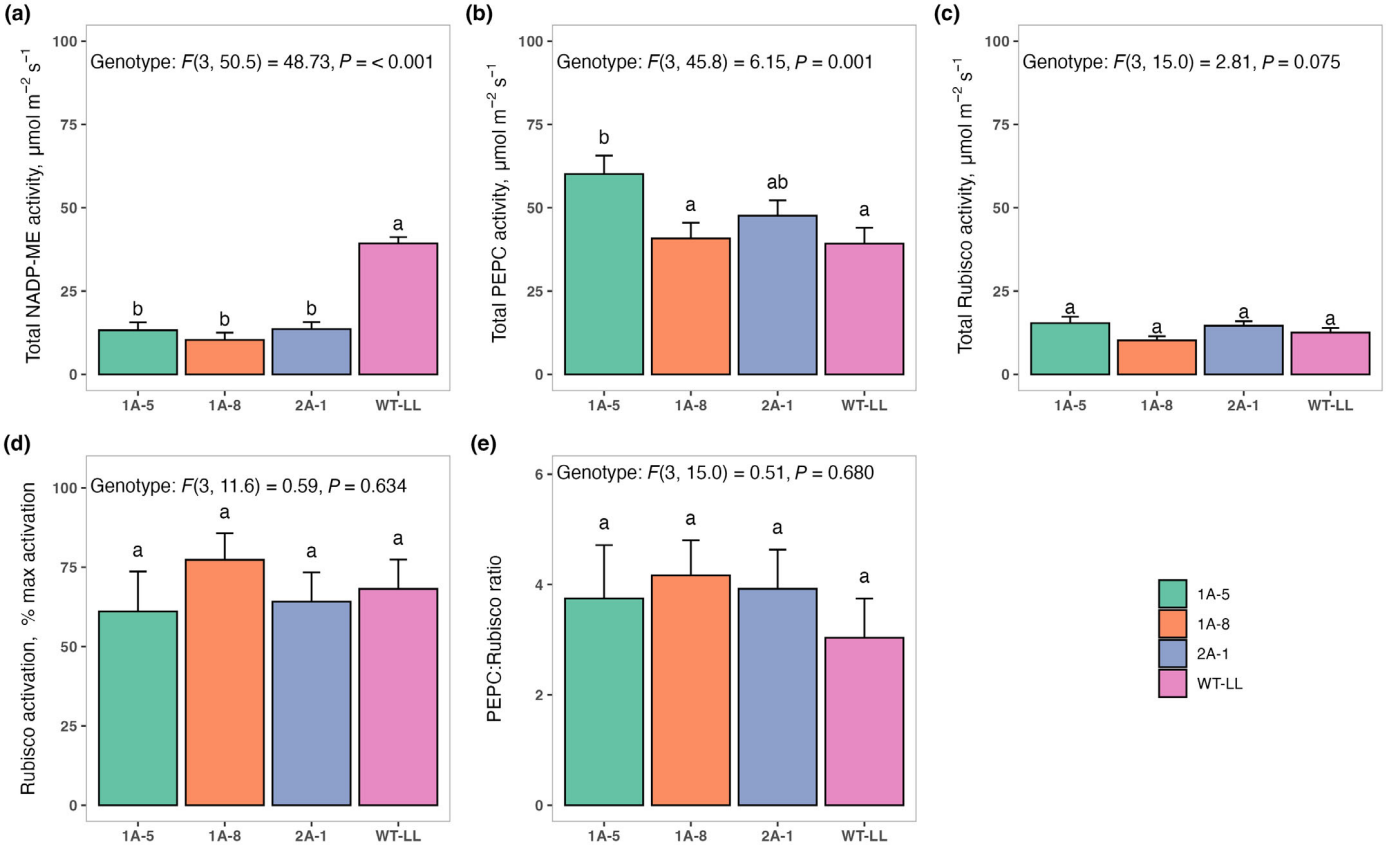
NADP‐malic enzyme (NADP‐ME), phosphoenol pyruvate carboxylase (PEPC), and Rubisco activity in wild‐type (WT) plants of *Flaveria bidentis* and plants expressing an antisense construct against NADP‐ME. Measurements were performed on soluble protein extracted from youngest fully expanded leaves of 8‐wk‐old plants (*n* = 3–6). (a) NADP‐ME (malic enzyme) activity. (b) PEPC activity (c). Total Rubisco activity (d) Rubisco activation status. (e) PEPC : Rubisco ratio. Values represent the mean ± SE. Statistical analysis was conducted using linear mixed‐effects models with genotype as a fixed effect and biological replication as a random effect. Significant differences between genotypes were assessed using model‐estimated contrasts based on Tukey's Honestly Significant Difference test. Different letters indicate statistical significance at α = 0.05.

Homozygous transgenic *F. bidentis* T_3_ plants with reduced NADP‐ME activity via antisense were used for all the presented analyses (see the Materials and Methods section). The effect of the antisense construct on NADP‐ME protein content was confirmed by immunoblotting (Fig. 1). Immunolabeling of blotted proteins with an antibody against NADP‐ME (provided by Prof Rob Sharwood) showed bands at the predicted size (65 kDa) for WT plants grown under regular (HL) or low light (LL, 150 μmol m^−2^ s^−1^) (Fig. 1, left panel). By contrast, strongly diminished bands were observed for 1A‐5, 1A‐8, and 2A‐1 plants (Fig. 1), confirming the impact of the antisense construct on NADP‐ME protein abundance. To assess the impact of the decrease in NADP‐ME protein content, total NADP‐ME enzyme activity was assessed using a spectrophotometric assay. Total leaf activity of NADP‐ME was significantly reduced (*P* < 0.001) in T_2_ homozygous populations of antisense *F. bidentis* plants (Fig. 2a). On average, NADP‐ME activity in the antisense lines was 12.4 ± 0.9 μmol m^−2^ s^−1^, compared to WT, which was 39.3 ± 2.7 μmol m^−2^ s^−1^, or *c*. 68% reduction in total NADP‐ME leaf activity. The reductions in NADP‐ME activity in the T_2_ antisense lines varied from 66 to 73%, which is less variable than the previously published values in the segregating T_1_ populations (Pengelly *et al*. 2012).

### Decreasing NADP‐ME activity does not significantly affect other key carbon assimilation enzymes

**Fig. 3:**
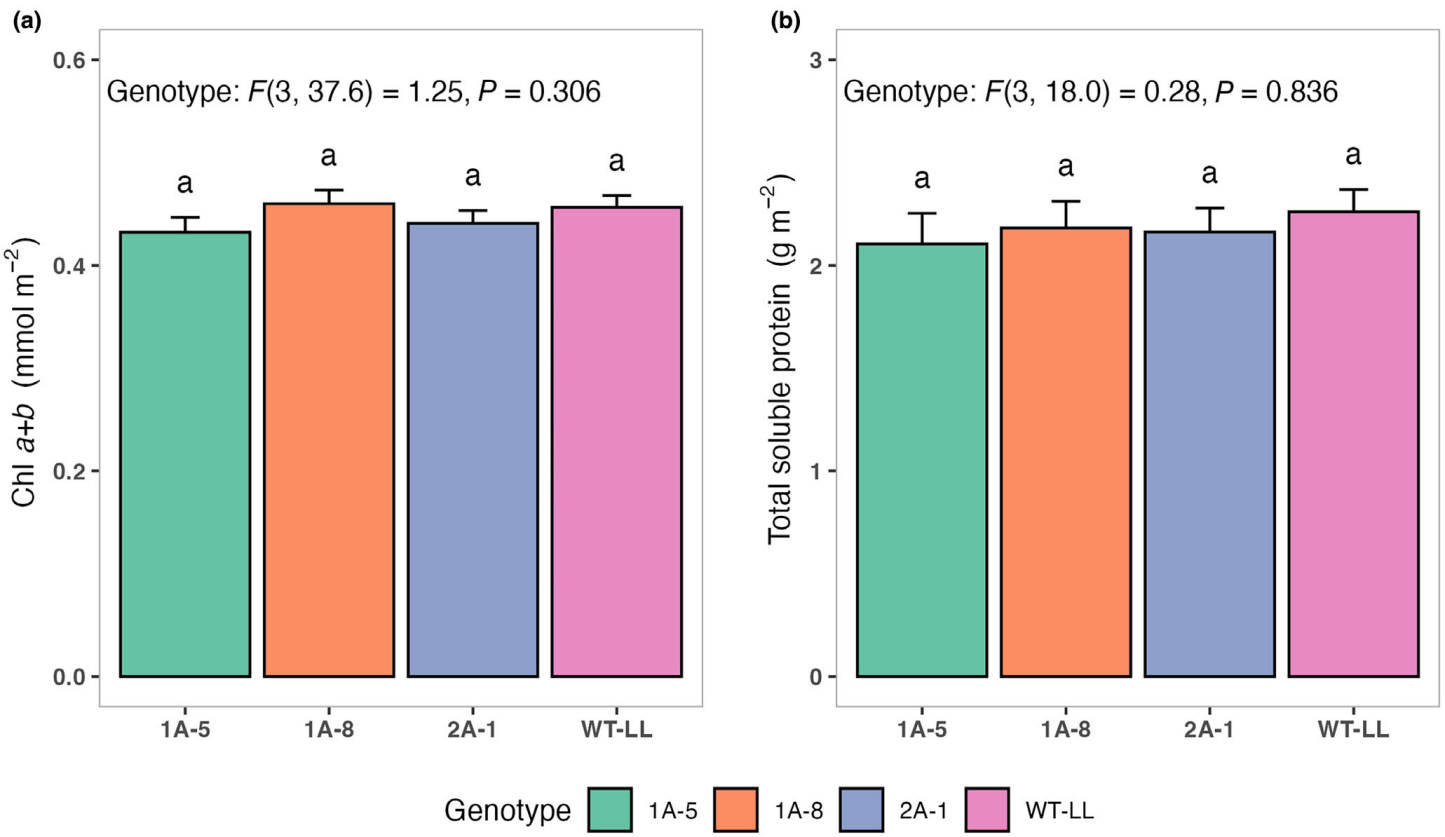
Chl and total protein content in wild‐type (WT) plants of C_4_ *Flaveria bidentis* and plants expressing an antisense construct against NADP‐malic enzyme. Total Chl (a) and total soluble protein (b) contents were measured spectrophotometrically from crude leaf extracts in 80% EtOH of 8‐wk‐old plants. Protein content was estimated against Bovine Serum Albumin standards using Bradford assay. Values represent the mean ± SE of *n* = 4–6. Statistical analysis was conducted using linear mixed‐effects models with genotype as a fixed effect and biological replication as a random effect.

To assess whether the strong reduction NADP‐ME activity influenced other key carbon assimilation enzymes, we measured PEPC and Rubisco activities in the same leaves, and extended the analysis to include Chl content and TSP. While genotype significantly affected PEPC activity (*P* < 0.0001), only 1A‐5 differed significantly from the WT control; thus, this did not appear to be a common property of NADP‐ME downregulation (Fig. 2b). Rubisco activity (Fig. 2c) and Rubisco activation state (Fig. 2d) did not show significant genotype effects (*P* = 0.075 and *P* = 0.634, respectively) and neither did PEPC : Rubisco ratios (*P* = 0.680). Similarly, no significant genotype effects were detected for total Chl content (*P* = 0.200; Fig. 3a) and TSP (*P* = 0.842; Fig. 3b).

### Operating intercellular (CO_2_ ) is significantly increased in NADP‐ME antisense lines of *F. bidentis*

**Fig. 4:**
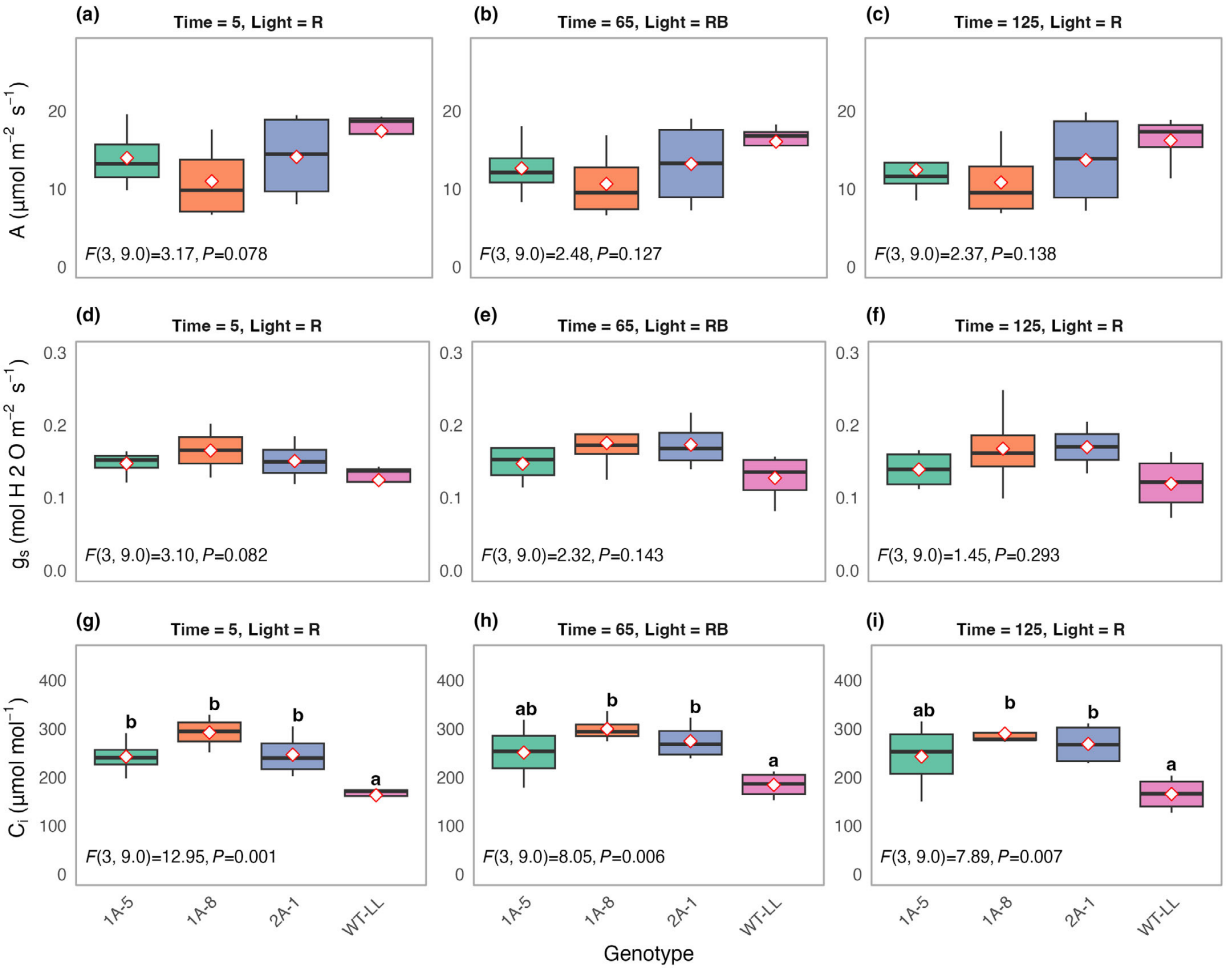
Stomatal conductance and intercellular CO_2_ concentration at two spectral light conditions in wild‐type (WT) plants of C_4_ *Flaveria bidentis* and plants expressing an antisense construct against NADP‐malic enzyme. CO_2_ assimilation rate (A; a, b, c), Stomatal conductance (*g*_s_; d, e, f), and intercellular CO_2_ concentration (C_i_; g, h, i) in WT‐LL and three antisense lines of C_4_ *F. bidentis* at 5, 65, and 125 mins under specified light conditions. Boxes show interquartile range, horizontal lines show median and whiskers show data range. Full timeseries are provided in Supporting Information Fig. S3. Significant differences in A, *g*_s_, and C*i* were determined using model‐estimated means with Tukey's Honestly Significant Difference adjustment at α = 0.05. Within each C_i_ (g–i) time point panel, box plots sharing the same letter are not significantly different. Diamonds indicate group means (*n* = 4).

To find out whether the decrease in NADP‐ME activity in antisense lines affected stomatal movements, *g*_s_ was assessed both under 100% red light and a mixture of 75% red and 25% blue light (Figs 4a–c, S3). Blue light did not have a significant effect on stomatal opening regardless of genotype. There was also no significant effect of genotype on the overall mean *g*_s_ throughout the experiment (mixed model analysis, *P* = 0.18), although the levels in WT plants were slightly lower at 0.125 ± 0.017 mol H_2_O m^−2^ s^−1^ than any of the mutants (1A‐5 = 0.146 ± 0.011 mol H_2_O m^−2^ s^−1^; 1A‐8 = 0.168 ± 0.021 mol H_2_O m^−2^ s^−1^, and 2A‐1 = 0.165 ± 0.015 mol H_2_O m^−2^ s^−1^). The analysis of C_i_, on the other hand, showed a significant genotype effect, with significantly higher values in antisense lines than in the WT control (Fig. 4d–f; *P* < 0.05).

### *g*_s_ is significantly increased in NADP‐ME antisense lines when measured at WT C_i_

**Fig. 5:**
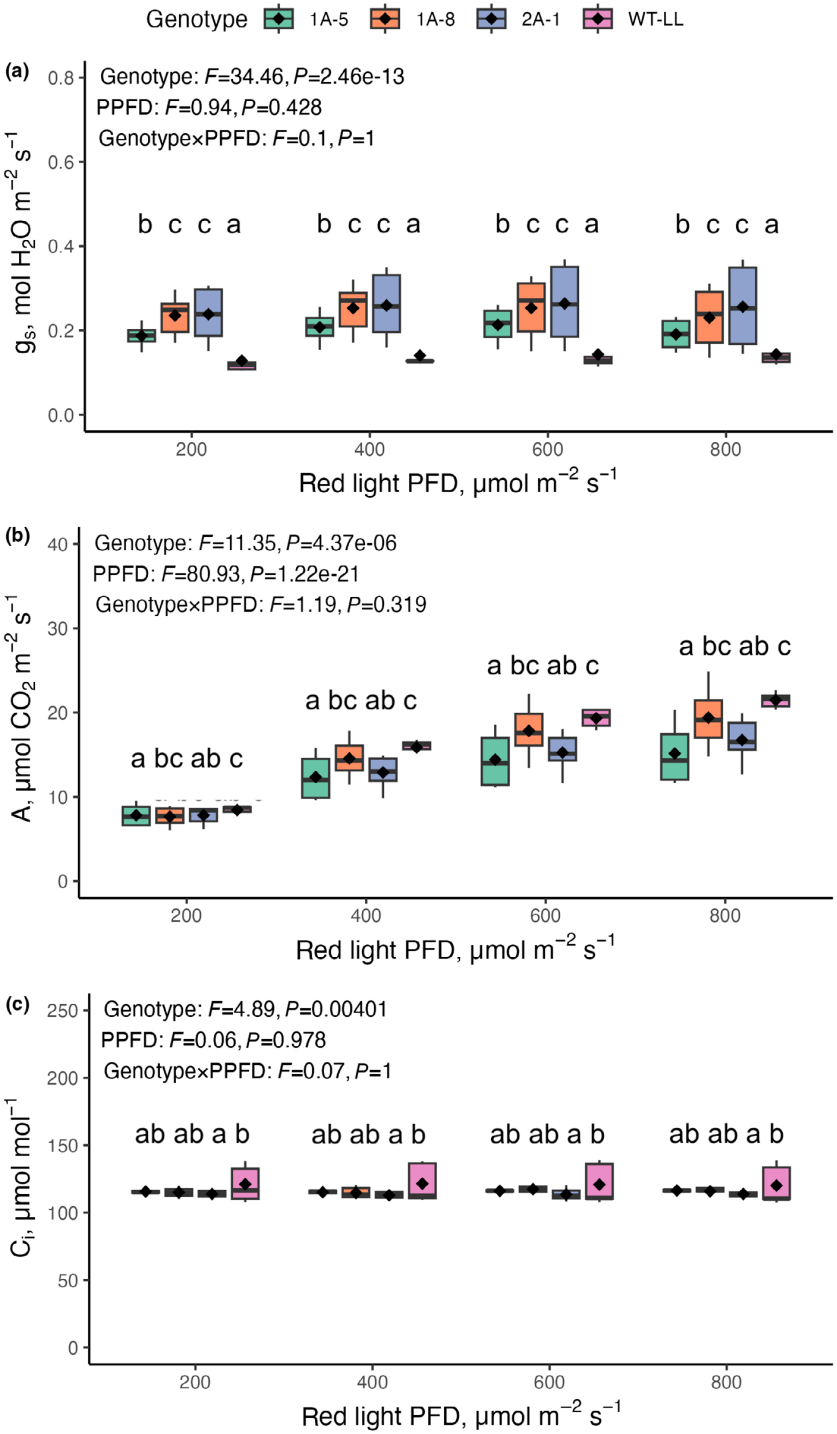
Stomatal conductance in response to varying light intensity, while keeping C_i_ constant in wild‐type (WT) plants of C_4_ *Flaveria bidentis* and plants expressing an antisense construct against NADP‐malic enzyme (NADP‐ME). Response of stomatal conductance (*g*_s_; a), CO_2_ assimilation rate (*A*_n_; b), and intercellular CO_2_ concentration (Cᵢ; c) in WT and independent NADP‐ME antisense lines of C_4_ *F. bidentis* were measured across increasing red light photon flux densities at a common Cᵢ of 120 μmol CO_2_ mol^−1^, matching the WT C_i_. Boxes show interquartile range, horizontal lines show median and whiskers show data range. Each box plot represents 4 to 6 biological replicates, with means indicated by black diamonds. Statistical groupings (letters) reflect differences among genotypes as determined by a linear mixed‐effects model accounting for replicate effects and evaluated across all light levels.

While *g*_s_ under either R or RB light did not vary between the NADP‐ME antisense lines and WT control plants, C_i_ during the measurements was markedly increased in the antisense lines and may have suppressed their level of *g*_s_. Therefore, a follow‐up experiment was carried out in which C_i_ was kept constant at 120 mol CO_2_ mol^−1^, the operating concentration in the WT plants, while incident red light intensity was varied (Fig. 5).

When incident red light intensity was reduced from 800 to 200 μmol photons m^−2^ s^−1^, the overall *g*_s_ for the antisense lines did not change and, ranging from 0.230 ± 0.020 mol H_2_O m^−2^ s^−1^ to 0.225 ± 0.014 mol H_2_O m^−2^ s^−1^. WT *g*_s_ was also constant across this range of light intensity but showed lower values at 0.151 ± 0.0127 mol H_2_O m^−2^ s^−1^ to 0.131 ± 0.0123 mol H_2_O m^−2^ s^−1^ (Fig. 5a). As a result, the interaction effect between genotype and light intensity was not significant (*P* = 0.9996), nor was the effect of light intensity (*P* = 0.4280). However, the effect of genotype on *g*_s_ was significant (*P* < <0.001), with significantly higher values in the three antisense lines relative to WT (*P* < 0.05).

Not surprisingly, CO_2_ assimilation rate responded significantly to the intensity of red light in all genotypes (*P* < 0.001; Fig. 5b). Although the effect of light intensity was highly significant, the interaction between genotype and light level was not (*P* = 0.319), suggesting that antisense and WT plants responded similarly to increasing red light, despite differing baseline assimilation capacities.

### Stomatal density and size are altered in NADP‐ME antisense lines

**Fig. 6:**
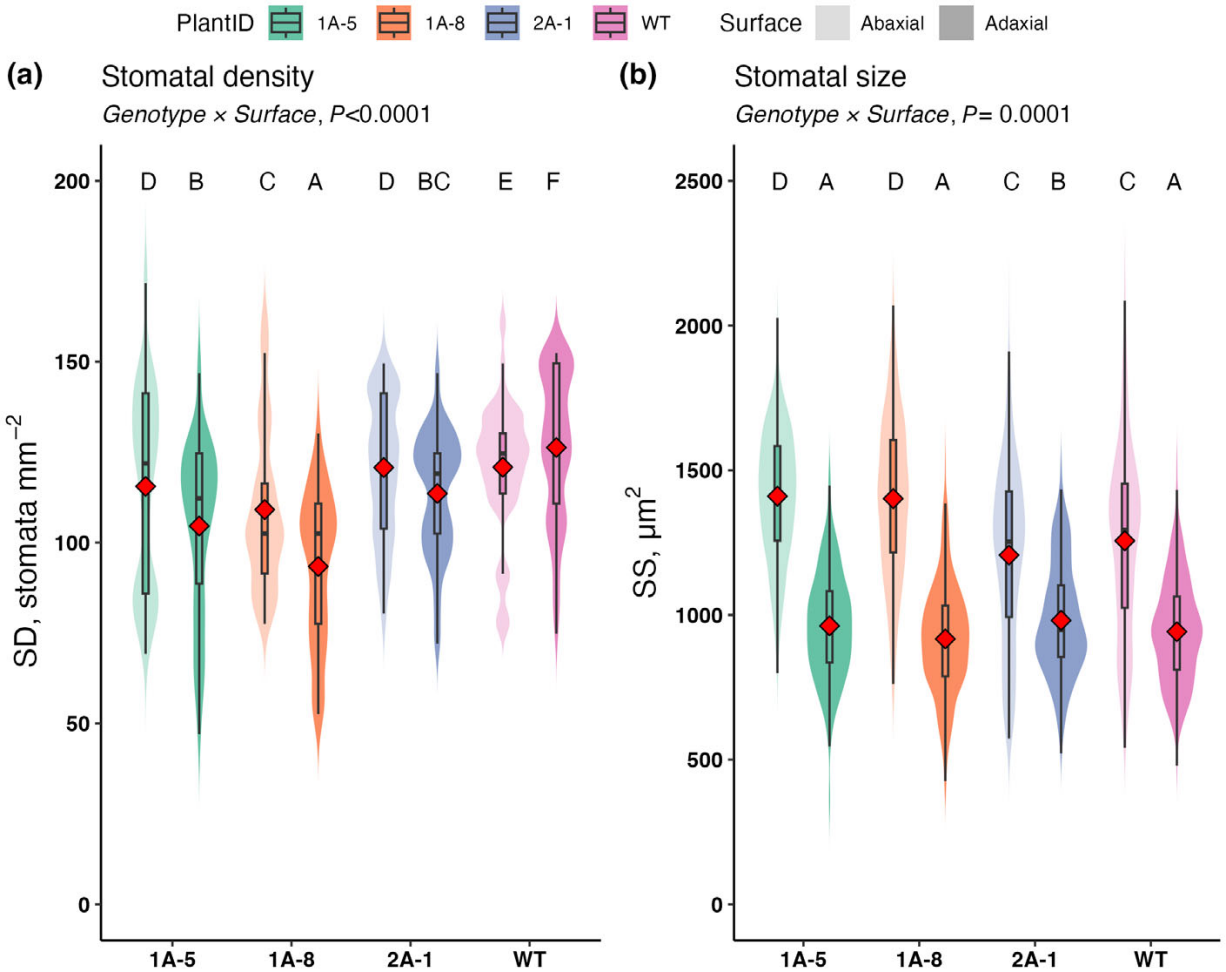
Stomatal density (SD) and size in wild‐type (WT) plants of C_4_ *Flaveria bidentis* and plants expressing an antisense construct against NADP‐malic enzyme (NADP‐ME). SD and size across different mutant lines and WT plants. (a) SD (stomata mm^−2^) and (b) stomatal size (SS, μm^2^) measured on both abaxial and adaxial leaf surfaces in WT and mutant lines (1A‐5, 1A‐8, 2A‐1). For each line, four random microscopic fields of view were collected from both the adaxial and abaxial surfaces of 3–5 independent biological replicates, representing more than 300 individual stomata per genotype. Each violin plot shows the distribution of values per biological replicate, with overlaid boxplots indicating median and interquartile range. Red diamonds represent the mean value for each group. Data were analyzed using a linear mixed model with Genotype, Surface (adaxial vs abaxial), and their interaction (Genotype × Surface) as fixed effects, and biological replicate as a random effect. Estimated marginal means were compared using *emmeans* with a Sidak correction for multiple comparisons (α = 0.05). Letters above violin plots indicate groups that are significantly different; groups not sharing a letter are significantly different at *P* < 0.05.

To determine whether the *g*_s_ differences corresponded to any differences in stomatal patterning or morphology, SD and SS were assessed. As observed previously in WT *F. bidentis* (Bernardo *et al*., 2023), SD varied between leaf surfaces (*P* < 0.0001) with higher SD and larger stomatal size in the abaxial layer than in the adaxial layer (Fig. 6). SD was slightly reduced in the NADP‐ME antisense lines relative to WT, particularly on the adaxial surface, while stomatal size was slightly increased on the abaxial surface in two of the antisense lines (1A‐5 and 1A‐8). Nevertheless, neither of these minor differences seems to be consistent with the observed increase in stomatal conductance, which instead seems to be a consequence of altered CO_2_ sensitivity of stomatal aperture changes.

## Discussion

This study examined the effect of antisense reduction of NADP‐ME activity, the major C_4_ acid decarboxylase in the C_4_ species *F. bidentis*, on stomatal conductance. Here, perturbation of the C_4_ cycle provided a means to investigate coordination between photosynthesis and stomatal conductance in plants with perturbed carbon assimilation while not directly targeting enzymes in the CBB cycle. Antisense suppression of NADP‐ME reduced enzyme activity by 66–73% across all lines, yet only moderately decreased light‐saturated net CO_2_ assimilation (*A*_n_) by 17 to 35%. Despite these significant reductions in *A*_n_, no corresponding decrease in stomatal conductance (*g*_s_) was observed.

Photosynthesis is well‐known to affect stomatal apertures by lowering C_i_ in leaves (Engineer *et al*., 2016). This has been referred to in literature as the C_i_‐dependent stomatal red‐light response (e.g. Taylor *et al*., 2024). When the negative impact of decreased photosynthesis on stomatal conductance was removed via assessment at common C_i_ of WT plants, NADP‐ME antisense plants had significantly higher *g*_s_, showing the importance of accounting for effects of the antisense construct on operational C_i_ when interpreting stomatal responses. In the following paragraphs, we discuss the relevance and wider implications of these findings.

### NADP‐ME antisense lines lack significant secondary effects

The antisense *F. bidentis* lines with reduced NADP‐ME activity used here were originally generated by Pengelly *et al*. (2012), who selected a partially differing subset of independent events for their studies and analyzed the segregating T_1_ generation. NADP‐ME activities and net assimilation rates of WT control plants found by Pengelly *et al*. (2012) were slightly higher than plants used here, potentially due to the low light intensity required to avoid stress responses in the homozygous antisense plants. The coordination between malate decarboxylation via NADP‐ME and RuBP carboxylation via Rubisco in the CBB cycle is critical to maintaining efficient carbon assimilation in C_4_ species and avoiding excessive leakiness (Kromdijk *et al*., 2014). Whereas Pengelly *et al*. (2012) reported slightly enhanced Rubisco and PEPC activity in the NADP‐ME antisense plants, this was not observed in this study (Fig. 2c). In addition, Pengelly *et al*. (2012) reported a slight increase in leaf nitrogen content in the antisense plants. Leaf nitrogen content was not evaluated in the present work, but total Chl and protein content were found to be unaffected by the antisense construct (Fig. 3). These slight differences could have resulted from a variety of reasons, including different growing conditions, as well as the use of two different transformation events, and the inclusion of both hemizygous and homozygous plants in the analysis by Pengelly *et al*. (2012). Nevertheless, Pengelly *et al*. (2012) observed decreased CO_2_ assimilation and increased C_i_/C_a_ across a range of light intensity, yet similar stomatal conductance in plants with relative NADP‐ME reduction similar to the lines analyzed here. While Pengelly *et al*. (2012) did not specifically measure stomatal conductance at common C_i_, measurements taken across a range of CO_2_ concentrations show that stomatal conductance under the same C_i_ likely increased in the NADP‐ME antisense plants, consistent with our observations.

### Evaluating stomatal conductance at common C_i_ may uncover ‘hidden’ phenotypes in plants with perturbed photosynthesis rates

Targeting CBB cycle enzymes has been a valuable approach to understand enzyme regulation and the coordination of C_3_ and C_4_ photosynthetic cycles (Raines, 2003, 2022; Lawson, 2009; Lawson *et al*., 2010; Lawson & Vialet‐Chabrand, 2019). In previous studies on C_3_ plants with antisense reductions in Rubisco, decreases of *c*. 35% in Rubisco content caused significant reductions in photosynthesis ranging from 50% in tobacco (Evans *et al*., 1994) to nearly complete limitation at higher irradiance; yet, these substantial declines in assimilation did not alter stomatal conductance significantly (Hudson *et al*., 1992; Stitt *et al*., 1992; Lauerer *et al*., 1993; Price *et al*., 1995). Similarly, in antisense *F. bidentis* plants with 34–75% reduction in NADP‐ME activity, there was a 30–50% decline in CO_2_ assimilation rates in lines with less than 40% NADP‐ME activity, particularly at high C_i_ concentrations; yet, stomatal conductance remained largely unaffected across a range of light intensities and CO_2_ levels (Pengelly *et al*., 2012). Collectively, these studies indicate a surprising lack of stomatal response to considerable reductions in assimilation rates in both C_3_ and C_4_ systems, highlighting that stomatal regulation can be uncoupled from photosynthetic capacity under certain genetic manipulations of photosynthetic enzymes.

When not accounted for, any increases in C_i_ arising from decreased (or increased) capacity for CO_2_ assimilation may lead to dampened (or enhanced) stomatal opening in any transgenic lines with altered photosynthesis. As demonstrated here, when removing the confounding effect of C_i_ by measuring at a common concentration, a significant increase in stomatal conductance in the antisense plants was uncovered, which was obscured by the opposing C_i_ effect. It seems plausible that similar effects may manifest more broadly if confounding effects of C_i_ were accounted for, or at the contrary, observed differences in stomatal conductance under common C_a_ can be removed under common C_i_ as demonstrated by observations on SBPase antisense tobacco plants by Lawson *et al*. (2008). The same study also reported a significant C_i_‐independent component, which is recognized to be part of the stomatal red‐light response (Messinger *et al*., 2006; Matrosova *et al*., 2015), accounting for *c*. 50% of stomatal red‐light responses in *Arabidopsis thaliana* (Taylor *et al*., 2024). However, in the current work, stomatal responses to changes in red light became negligible in both antisense mutants and WT *F. bidentis* plants when C_i_ was kept constant, in line with previous observations in WT *F. bidentis* (Bernardo *et al*., 2023), suggesting that alterations in this response are unlikely to underpin the observed increase in stomatal conductance in the antisense plants.

Keeping C_i_ constant during gas exchange measurements is relatively trivial but time‐consuming, requiring iterative adjustment of C_i_ via C_a_ using the instrument CO_2_ control settings while counteracting stomatal responses to mitigate the effect of each change in C_a_ on C_i_. However, control of C_i_ via C_a_ means that to keep C_i_ constant, C_a_ must vary, thus potentially confounding the stomatal response if guard cells are able to sense C_a_. The effect of CO_2_ on stomatal movements was described as early as Darwin (1898) and Linsbauer (1916), with Heath & Russell (1954) later recognizing the relevance of intercellular CO_2_ in this response. Using a split‐chamber approach, Mott (1988) demonstrated that stomata exclusively sensed C_i_ in leaves of two amphistomatous species by showing that stomatal responses to C_i_ were not affected by varying concentrations in air or at the leaf surface. Since this seminal work, the consensus has been that guard cells exclusively sense C_i_. However, the fact that CO_2_ sensing is guard cell autonomous (Engineer *et al*., 2016) and that substantial species variation appears to exist in stomatal sensitivity to CO_2_ (Liang *et al*., 2023), suggests that a contribution of the CO_2_ concentration inside the pore in some species may not be ruled out *a priori*, as suggested previously by von Caemmerer *et al*. (2004). Nevertheless, considering the elevated C_i_/C_a_ as a general feature of most transgenic plants with CBB cycle or C_4_ cycle impairments, even with a moderate contribution of CO_2_ in the pore, the average CO_2_ sensed by stomata is likely increased, with the suppressing effect of stomatal opening as a result. Based on this elevation in the concentration sensed by stomata, experiments should also account for potential acclimatory decreases in SD to elevated C_a_ such as seen in species grown at elevated CO_2_ (Hetherington & Woodward, 2003) and across geological timescales with contrasting atmospheric compositions (Woodward, 1987). Alternatively, use of inducible constructs (Schlücking *et al*., 2013) may allow a more precise way to study the direct impact of mutations on stomatal responses by removing the potentially confounding impact of acclimatory responses to constitutive knockdowns.

### The molecular mechanism underlying increased *g*_s_ at constant C_i_ in antisense NADP‐ME plants remains unresolved

Our observations in C_4_ cycle mutants of *F. bidentis* confirm those of several previous studies on CBB cycle mutants, showing that the negative impacts of decreased CBB cycle or C_4_ cycle enzyme expression on CO_2_ assimilation are independent of stomatal function *per se*. Indeed, the apparent coordination observed in unperturbed plants does not reflect a direct link between photosynthesis and stomatal conductance, consistent with previous conclusions (for a review, see Lawson *et al*., 2014). Nevertheless, photosynthesis affects stomatal conductance via its effect on C_i_ (Lawson, 2009), which should generally lead to a decline in genetic mutant plants with higher C_i_. The fact that this is often not observed may imply that the prevailing stomatal conductance in these mutants is not determined by optimization of carbon gain over water loss, but instead reflects stomatal acclimation to the prevailing growth conditions to support alternative functions of transpiration, such as bulk water movement to drive nutrient uptake and translocation (Matimati *et al*., 2013). While this can be achieved via adjustment of stomatal size and patterning (Casson & Gray, 2008), this was not the case here (Fig. 6). Instead, the fact that stomata in the antisense plants opened more than WT plants under common C_i_ but not under ‘growth C_i_’ suggests that such acclimation may involve an adjustment of the molecular players involved in sensing and guard cell turgor responses to high C_i_. These could include β‐CARBONIC ANHYDRASE 1 and 4 (CA1 and CA4), which help to produce bicarbonate that activates S‐type anion channels (Xue *et al*., 2011); the HIGH LEAF TEMPERATURE 1 (HT1) protein kinase (Hashimoto *et al*., 2006), which suppresses the high CO_2_ stomatal closing response by post‐translational suppression of OPEN STOMATA 1 (OST1) activity (Horak *et al*., 2016), which is a positive regulator of the SLOW ANION CHANNEL ASSOCIATED 1 anion channel (Brandt *et al*., 2012); the RESISTANT TO HIGH CO2 MATE‐type transporter, which may act upstream of HT1 to suppress its activity under high CO_2_ (Tian *et al*., 2015), although phenotypes were not consistently repeatable (Toldsepp *et al*., 2018); and finally, mitogen‐activated protein kinases MPK4 and MPK12, which mediate stomatal CO_2_ responses (Toldsepp *et al*., 2018) via inhibition of HT1 activity (Horak *et al*., 2016). It seems plausible that small acclimatory adjustments in expression or post‐translational modification could give rise to the observed phenotypes.

An alternative hypothesis could be that guard cell malate pools are affected by the antisense construct. Malate is involved in stomatal opening as a counterion for potassium accumulation to keep membrane potential by balancing charge (Allaway, 1973; Outlaw Jr & Lowry, 1977). Work by Cousins *et al*. (2007) on the C_4_ species *Amaranthus edulis* showed that strong antisense reduction of PEPC to 3% of WT levels led to slower stomatal opening and lower steady state conductance, which was suggested to stem from slower and less abundant malate formation in guard cells. Based on recent single nucleus and single‐cell RNAseq data on *F. bidentis* (Sun *et al*., 2025), it is clear that while the antisense construct was raised against the C_4_ NADP‐ME isoform (FBID‐00003378; Fig. S4A), there is 89.9% sequence conservation of the antisense fragment with the main chloroplastic isoform expressed in guard cells (F‐bidentis‐14‐000158; Fig. S4B). It is therefore plausible that the antisense construct against NADP‐ME used in the present work also decreased NADP‐ME activity in the guard cells. If so, the resulting alteration in guard cell malate metabolism may have affected *g*_s_ in the opposite way compared to the PEPC antisense observations by Cousins *et al*. (2007). During stomatal closure, malate is decarboxylated to pyruvate by NADP‐ME, which is then completely oxidized in the mitochondrial tricarboxylic acid cycle. Alternatively, malate can be converted to PEP via the combined action of NAD^+^‐dependent MDH and PEP carboxykinase (PEPCK) (Santelia & Lawson, 2016). Thus, if NADP‐ME activity in guard cells decreased in antisense lines, accumulated malate during stomatal opening may not be as rapidly metabolized, which could give rise to increased stomatal opening and slower stomatal closure, in line with the observed higher *g*_s_ at common C_i_.

### Conclusion

This study focused on the consequences of a disrupted C_4_ cycle on the regulation of *g*_s_. The results showed that at common C_a_, NADP‐ME antisense lines of C_4_ *F. bidentis* had similar stomatal conductance but a higher operational C_i_ than WT, whereas measurements at a common C_i_ showed an increase in *g*_s_ in the antisense lines. These findings are most consistent with altered guard cell C_i_ sensitivity in the antisense lines. We postulate that altered CO_2_ sensing in guard cells may provide a molecular explanation for the increase in *g*_s_ in common C_i_.

## Competing interests

None declared.

## Author contributions

JK conceptualized the study. ELB managed plant growth and maintenance, performed preliminary testing on wild‐type and mutant plants, conducted gas exchange experiments and collected, processed and analyzed the data. ELB and CRS carried out the enzyme activity assays and Chl content determination. TS provided cell‐specific expression data for NADP‐ME isoforms. ELB and JK drafted the initial manuscript. All authors reviewed and approved the final manuscript.

## Disclaimer

The New Phytologist Foundation remains neutral with regard to jurisdictional claims in maps and in any institutional affiliations.

## Supporting information


**Fig. S1** Bleaching of antisense NADP‐ME plants under 350 μmol m^−2^ s^−1^ light intensity is mitigated by 150 μmol m^−2^ s^−1^ light intensity.
**Fig. S2** Representative image of wild‐type *Flaveria bidentis* plants and three lines carrying independent insertions of the NADP‐ME antisense construct.
**Fig. S3** Time course of *g*_s_ in WT and NADP‐ME antisense lines of C_4_ *Flaveria bidentis*.
**Fig. S4** Expression of different NADP‐ME isoforms across contrasting cell types.
Please note: Wiley is not responsible for the content or functionality of any Supporting Information supplied by the authors. Any queries (other than missing material) should be directed to the *New Phytologist* Central Office.

## Data Availability

All data that support the findings of this study are available in the main text and Figs S1–S4 of this article.
