# Supplementary material for: Antisense reduction in NADP‐ME in the C4 species Flaveria bidentis alters stomatal sensitivity to intercellular [CO2 ]
Source: New Phytol. 2026 Feb 18;250(3):1585–97. doi: 10.1111/nph.71012 (PMC13062702; doi:10.1111/nph.71012)
Supplement: Supplementary file 1 — Fig. S1 Bleaching of antisense NADP‐ME plants under 350 μmol m−2 s−1 light intensity is mitigated by 150 μmol m−2 s−1 light intensity. Fig. S2 Representative image of wild‐type Flaveria bidentis plants and three lines carrying independent insertions of the NADP‐ME antisense construct. Fig. S3 Time course of g s in WT and NADP‐ME antisense lines of C4 Flaveria bidentis. Fig. S4 Expression of different NADP‐ME isoforms across contrasting cell types. Please note: Wiley is not responsible for the content or functionality of any Supporting Information supplied by the authors. Any queries (other than missing material) should be directed to the New Phytologist Central Office. [file NPH-250-1585-s001.docx]

# **New Phytologist Supporting Information**

# **Article title**:

# Antisense reduction in NADP-ME in the C_4_ species *Flaveria bidentis* alters stomatal sensitivity to intercellular [CO_2_]

**Authors**:

Emmanuel L. Bernardo^1,2^, Cristina Rodrigues Gabriel Sales^1^, Tianshu Sun^1^, Johannes Kromdijk^1,#^

Article acceptance date: 26 January 2026


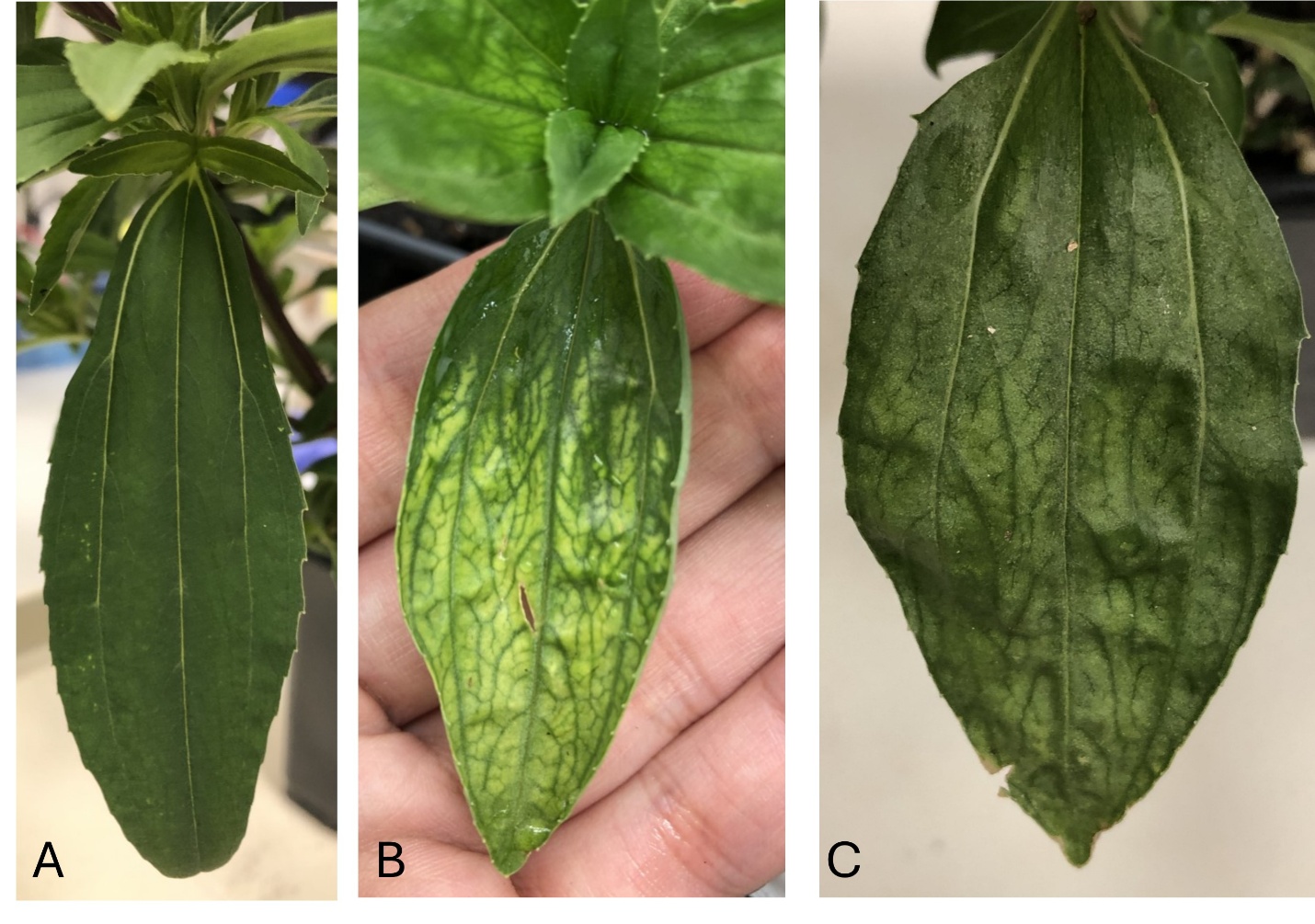


**Supplemental Figure 1** Bleaching of antisense NADP-ME plants under 350 µmol m^-2^ s^-1^ light intensity is mitigated by 150 µmol m^-2^ s^-1^ light intensity. A) Wildtype leaf grown under 350 µmol m^-2^ s^-1^, B) NADP-ME antisense leaf grown under 350 µmol m^-2^ s^-1^, C) NADP-ME antisense leaf grown under 150 µmol m^-2^ s^-1^


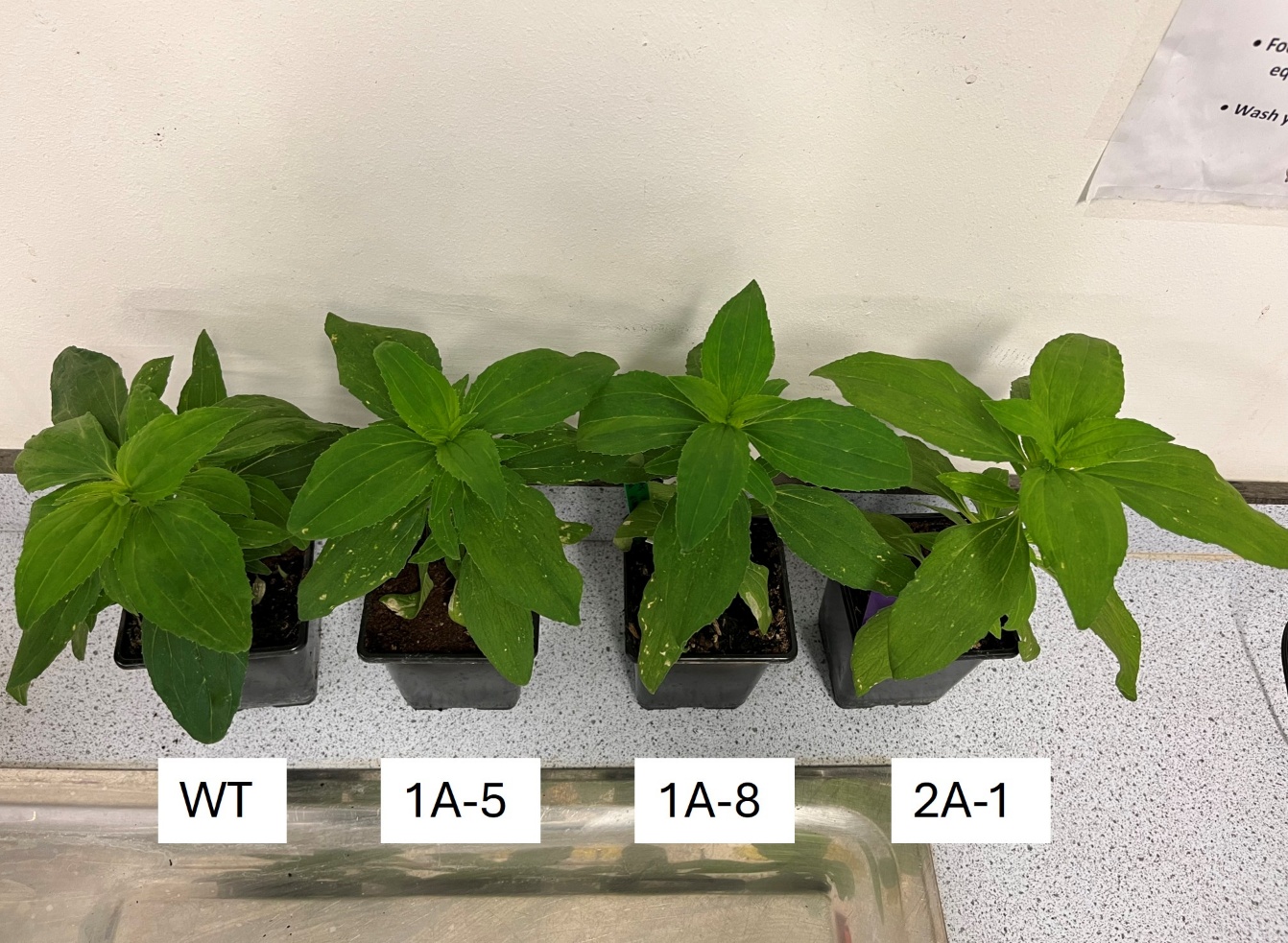


**Supplemental Figure 2** Representative image of wildtype *Flaveria bidentis* plants and three lines carrying independent insertions of the NADP-ME antisense construct.


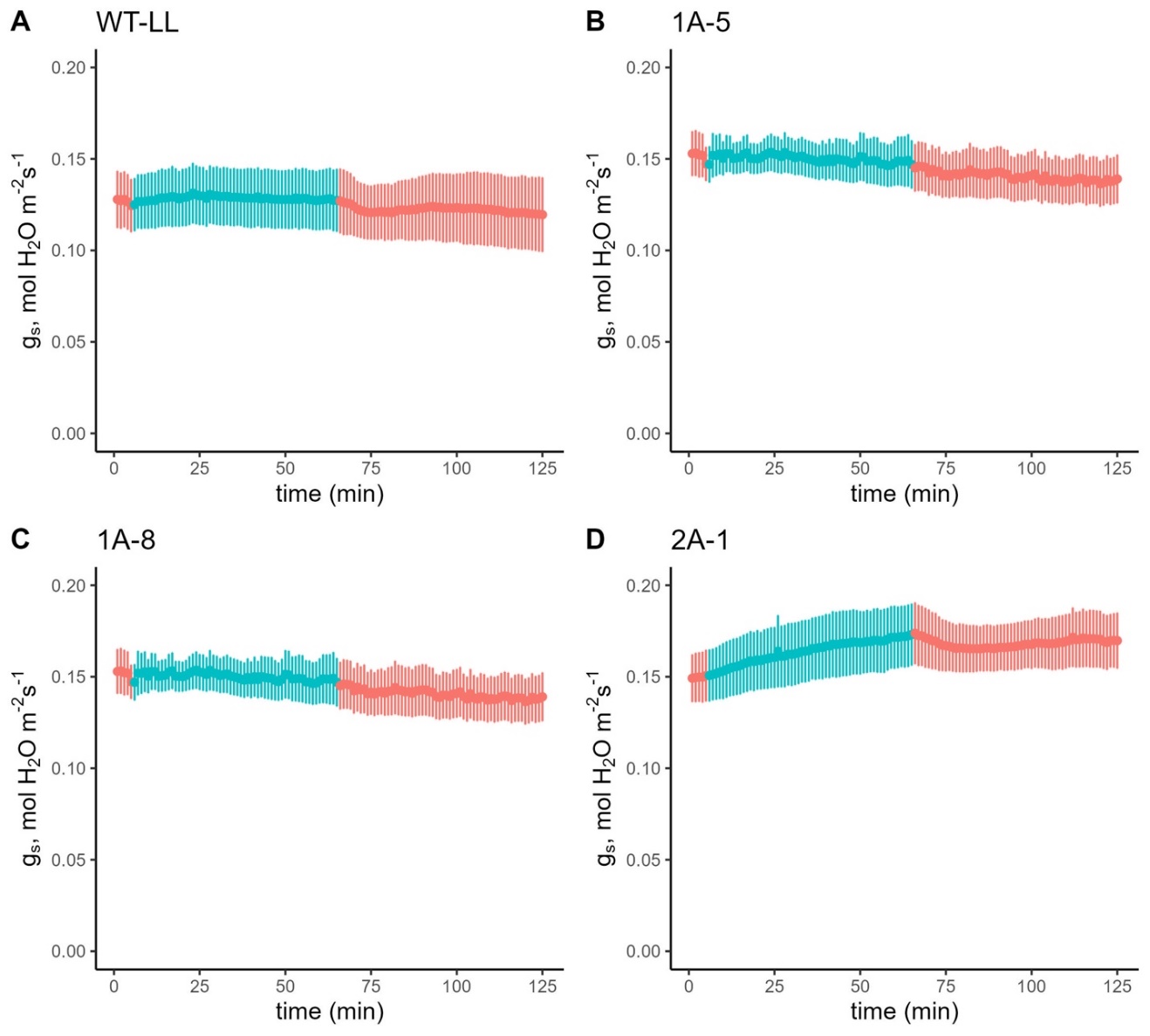


**Supplemental Figure 3.** Time course of g_s_ in WT (A) and antisense lines of C_4_ *F. bidentis* (B, C, and D). The leaves were initially acclimated in 100% red light (red symbols) until steady state was achieved. Subsequently, the light environment was switched to 75% red + 25% blue (blue symbols) while maintaining an intensity of 500 μmol m^-2^ s^-1^. In each case, leaves were acclimated to the new light condition for 60 min before returning them back to the original condition for another 60 min before terminating the experiment. The light conditions were reversed at t_6_ and t_66_. Reference CO_2_ was maintained at 410 μmol mol^-1^, block temperature was kept at 25º C and the mean leaf-to-air VPD was 1.2 kPa. Data points represent the mean ± s.e.m. (n=4).

**
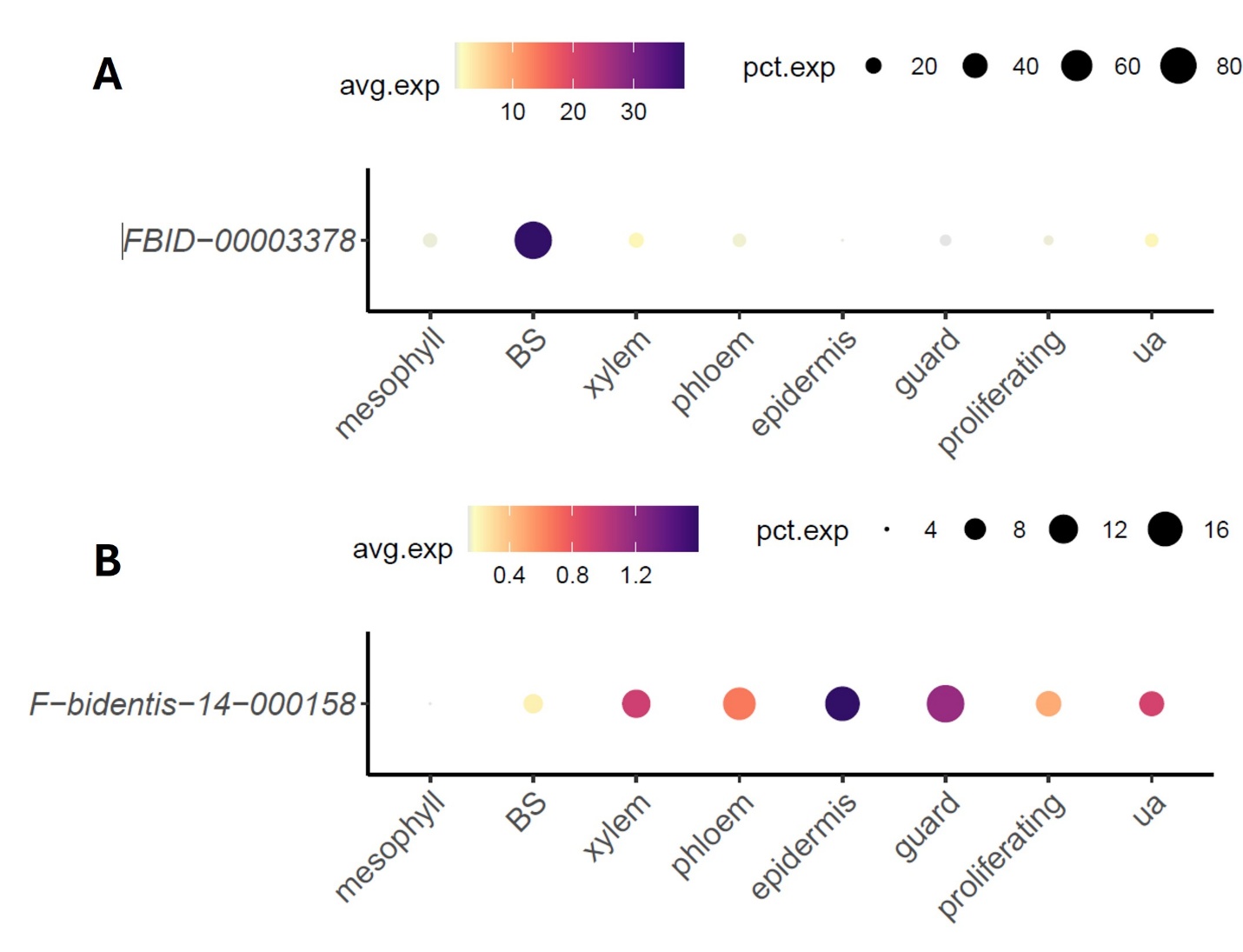
**

**Supplemental Figure 4.** Expression of different NADP-ME isoforms across contrasting cell types. Dot plots show the expression of the C4 isoform of NADP-ME (FBID-00003378; A) and the most strongly expressed chloroplastic NADP-ME isoform in guard cells (F-bidentis-14-000158; B) across cell types in the *F. bidentis* single-cell RNAseq dataset by Sun et al. (2025). The colour scale indicates the average expression level within each cell type, calculated from library-size–normalised and log1p-transformed expression values for individual cells. The dot size reflects the percentage of cells expressing the gene. BS = bundle sheath cells, guard = guard cells, ua = unassigned.
